# Diagnostic performance of APRI and FIB-4 for confirming cirrhosis in Indonesian HIV/HCV co-infected patients

**DOI:** 10.1186/s12879-020-05069-5

**Published:** 2020-05-25

**Authors:** Evy Yunihastuti, Bramantya Wicaksana, Andrian Wiraguna, Ainum Jhariah Hidayah, Fhadilla Amelia, Veritea Natali, Alvina Widhani, Andri Sanityoso Sulaiman, Juferdy Kurniawan

**Affiliations:** 1 HIV integrated services, Cipto Mangunkusumo Hospital, Jakarta, Indonesia; 2 Department of Internal Medicine, Faculty of Medicine Universitas Indonesia, Cipto Mangunkusumo Hospital, Diponegoro, Jakarta, 71 Indonesia

**Keywords:** APRI, FIB-4, Transient elastography, HIV, HCV

## Abstract

**Background:**

After successful of antiretroviral therapy, highly effective direct acting antiviral (DAA) make HCV elimination reasonable in HIV/HCV co-infected patients. However, in achieving this target, there are still barriers to start DAA treatment, particularly in the area of liver fibrosis assessment that determine the duration of therapy. We aimed to assess the diagnostic performance of APRI and FIB-4 for diagnosing cirrhosis in HIV/HCV co-infected patients using hepatic transient elastography (TE) as gold standard.

**Method:**

This is a retrospective study on HIV/HCV co-infected patients who concomitantly performed hepatic TE measurement, APRI, and FIB-4 evaluation before HCV treatment initiation at a tertiary hospital in Jakarta from 2014 to 2019. Sensitivity, specificity and diagnostic accuracy of indirect biomarkers for liver stiffness measurement (LSM) ≥ 12.5 kPa was determined by receiver operator characteristics curves.

**Results:**

223 HIV/HCV co-infected patients on stable antiretroviral therapy were included, of whom 91.5% were male with mean age of 37 (SD 5) years. Only 28.7% of patients were classified as cirrhosis (F4). Using TE as gold standard (≥12.5 kPa), the low threshold of APRI (1) had specificity 95%, sensitivity 48.4%, correctly classified 81.6% of patients, with moderate performance, AUC at 0.72 (95% CI 0.63–0.80). The optimal cut-off of FIB-4 was 1.66 [specificity 92.5%, sensitivity 53.1%, AUC at 0.73 (95% CI 0.65–0.81)] and correctly classified 81.1% of the patients.

**Conclusion:**

APRI score ≥ 1 and FIB-4 score ≥ 1.66 had moderate performance with high specificity in diagnosing cirrhosis. These biochemical markers could be used while TE is not available.

## Background

Hepatitis C virus (HCV infection) is one of global health problem that can increase morbidity and mortality in HIV-infected population. In 2017, about 71 million people living with chronic HCV infection globally, of which only 19% (13.1 million) knew their diagnosis. The estimated prevalence in South-East Asia was 0.5% with incidence 14.8 (95% CI 12.5–26.9) per 100,000 population in 2015 [1]. In Indonesia, HCV infection prevalence is estimated at 1%, of which 90% comes from intravenous drug user [2]. World Health Organization (WHO) recently released targets for HCV elimination by 2030, targeting 80% HCV-infected patients being treated [1]. A large gap between HCV-diagnosed patients and HCV-treated patients still exist to achieve this global target since only around 10% of HCV-infected patients in South East Asia have been treated [3]. Achieving global elimination of HCV need major increase in service all populations along the entire cascade of care, including testing, linkage to care, retention in care, treatment, chronic care and prevention of primary infection and reinfection [4]. In 2017, Indonesian government started free direct acting antiviral (DAA) program for HCV, mainly sofosbuvir and daclatasvir combination [2, 5]. This 12 weeks combination for non-cirrhotic patients and 24 weeks for cirrhotic-patients shown similar success rate for both HIV/HCV co-infected patients and HCV mono-infected patients [6]. Despite these facts, there are still many barriers to start HCV treatment, especially in defining liver fibrosis phase. Liver biopsy has been gold standard for liver fibrosis assessment for a long time. This invasive procedure carries some serious side effects and can only be done in specialized care. Transient elastography (TE) is a non-invasive method that measures liver stiffness using both ultrasound and low-frequency elastic waves to define liver fibrosis phase [7]. This method has been validated in viral hepatitis with equal performance in HCV and HIV/HCV co-infection [8]. However, it is not widely available in Indonesia and still carries a high-cost examination.

Several laboratory tests, scores, and indices have been developed for non-invasive prediction of cirrhosis (F4) in HCV mono-infected patients. Aspartate aminotransferase (AST)-to- platelet ratio (APRI), fibrosis-4 (FIB4), modified APRI, Forns, and Göteborg University Cirrhosis Index (GUCI) are based on routine laboratory parameters and readily available in clinical practice. The two most common and validated used are APRI and FIB-4. WHO ranked these two scores as preferred non-invasive markers of fibrosis in resource-limited setting [9].

Since the determination of liver cirrhosis significantly influences the decision for treatment duration of sofosbuvir and daclatasvir, optimal cut-off of APRI and FIB-4 to diagnose cirrhosis need to be defined in our HIV/HCV co-infected population. We aimed to assess the diagnostic performance of APRI and FIB-4 for diagnosing liver cirrhosis in Indonesian HIV/HCV co-infected patients using hepatic TE (Fibroscan®) as a gold standard.

## Methods

### Study design

A retrospective study on HIV/HCV co-infected patients who concomitantly performed hepatic TE measurement, APRI, and FIB-4 evaluation before HCV treatment initiation at HIV Integrated Clinic, Cipto Mangunkusumo Hospital, Jakarta between 2014 and December 2019. The demographic data, clinical, and laboratory data were collected from hospital medical record. Clinical data collected including body mass index (BMI), HBsAg seropositivity, and ART history. Laboratory data including HCV RNA, complete blood count (CDC), transaminase value, and estimated glomerular filtration rate (eGFR).

### Eligibility criteria

Inclusion criteria were infection with HCV defined as a positive HCV-RNA, HIV infection on stable antiretroviral treatment, and no prior HCV treatment. Exclusion criteria was hepatocellular carcinoma.

### Instrumental assessment

Liver fibrosis or cirrhosis was assessed using transient elastography (FibroScan 502®, Echosens, France) by trained operators according to the manufacturer’s protocol. The procedures of liver stiffness measurement (LSM) were patient in a supine position, then M probe was put in intercostal spaces on the right lobe. The value of LSM was expressed in kilopascal (kPa). Patients were recommended to do fasting before the examination. LSM values were included in this analysis with at least 10 valid measures, over 60% success rate and interquartile range (IQR) less than 30% of the median value of LSM [10]. The following cut-offs were used to stage the liver fibrosis are F0-F1 < 7.1 kPa, F2 (significant fibrosis) 7.1–9.4 kPa, F3 (advanced fibrosis) 9.5–12.4 kPa, and F4 (cirrhosis) ≥12.5 kPa [11].

The APRI score was calculated using the formula: [(AST level/ULN)/platelet count (10^9^/L)] × 100 [9]. The reference upper normal limit (ULN) for AST used in our hospital was 44 IU. While the FIB-4 score was using formula: age x AST/(platelet count x √ALT) [9].

### Statistical analysis

All statistical analysis were performed using STATA statistical package for Windows version 12.0 (Statacorp, USA). The description of socio-demographics, HIV characteristics, and HCV characteristics was performed using standard descriptive methods. Categorical variables were expressed as raw numbers and percentages and compared using the chi-square test, and continuous variables were described by means and standard deviation (normal distribution) or median and IQR 25–75% (non-normal distribution assessed by Kolmogorov-Smirnov test). The proportion of liver cirrhosis (F ≥ 4) was assessed as the number of patients with LSM of minimum 12.5 kPa and expressed with its 95% confidence interval. A two-sided *p* value < 0.05 was considered to be statistically significant.

Performances of APRI and FIB-4 in diagnosing cirrhosis were evaluated from Area Under Receiver-Operator Curves (AUROCs) using binomial method, considering TE as the gold standard. Using WHO hepatitis C guidelines, two thresholds for fibrosis (F ≥ 4) with APRI examined: 2 (high threshold) and 1 (low threshold) [9]. While the diagnosis of cirrhosis (F ≥ 4) with FIB-4 was examined as 1.94 and 3.25 cut-offs based on previous study in HIV/HCV co-infected patients [12]. To evaluate the diagnostic accuracy of available cut-offs of APRI and FIB-4, sensitivity, specificity, positive predictive value (PPV), negative predictive value (NPV), positive likelihood ratio (LR+), negative likelihood ratio (LR-) were estimated for each cut-off value. Accuracy was assessed by the percentage of patients that were correctly classified. In finding the novel diagnostic cut-offs for APRI and FIB-4 in our population, the ROCs were generated by plotting the relationship of the true positivity (sensitivity) and the false positivity (1-specificity) at various cut-off points of each score. We choose optimal cut-off point using to the highest rate of correctly classified patients.

## Results

### Patient characteristics

Of 223 study patients, 91.5% were male with a mean age of 37 (SD 5) years. All patients were under stable antiretroviral therapy (ART) medication, mostly efavirenz (EFV)-based regiment (43%) or nevirapine (NVP)-based regiment (42.2%). Median absolute CD4 cell count was 508 (IQR 306) cells/mm^3^ and the median duration of ART was 6.8 (IQR 4) years. Most of patients (93.3%) had history of intravenous drug use (IVDU) and 71.3% patients reported to have past alcohol consumption. Only 8 patients (3.6%) had all hepatitis B, hepatitis C, and HIV infections. All demographics and HIV characteristics were similar between cirrhotic and non-cirrhotic patients, except ART-regimen used (Table 1**).** We did not find any decompensated cirrhosis among 64 cirrhotic patients within recruitment phase (the data was not shown).

**Table 1:** Description of the study population according to the liver cirrhosis status

	Total (*n* = 223)	Non-cirrhosis (LSM < 12.5 kPa) (*n* = 159)	Cirrhosis (LSM ≥ 12.5 kPa) (*n* = 64)	*p* value
***Socio-demographic characteristics***				
Age, years, mean (SD)	37 (5)	37 (5)	38 (6)	0.376
Male, n(%)	204 (91.5)	142 (89.3)	62 (96.8)	0.068
BMI, kg/m^2^, mean (SD)	22.55 (3.80)	22.26 (3.56)	23.27 (4.27)	0.071
IVDU history, n(%)	208 (93.3)	148 (93.1)	60 (93.7)	0.857
Alcohol history, n(%)	159 (71.3)	114 (71.7)	45 (70.3)	0.836
HBsAg seropositivity, n(%)	8 (3.6)	7 (4.4)	1 (1.5)	0.303
***HIV characteristics***				
ART duration, years, median (IQR)	6.8 (4)	6.8 (5)	6.8 (2)	0.264
Current CD4, cells/mm^3^, median (IQR)	508 (306)	508 (333)	508 (215)	0.855
***ART used***				
2NRTI + EFV, n(%)	96 (43)	76 (47.7)	20 (31.2)	0.011
2NRTI + NVP, n(%)	94 (42.2)	64 (40.2)	30 (46.8)	
2NRTI + LPV/r, n(%)	33 (14.8)	19 (11.9)	14 (21.8)	
***HCV infection characteristics***				
HCV-RNA, log_10_, mean (SD)	6.12 (0.81)	6.15 (0.84)	6.06 (0.73)	0.453
Hemoglobin, g/dL, mean (SD)	13.8 (2.3)	13.9 (2.0)	13.5 (2.8)	0.201
Platelet, 10^3^/mL, median (IQR)	225 (77)	249 (78)	173 (72)	< 0.001
ALT, U/L, median (IQR)	55 (51)	50 (41)	66.5 (71)	< 0.001
AST, U/L, median (IQR)	41 (41)	37 (25)	71 (64)	< 0.001
eGFR (CKD-EPI), mL/min/1.73 m^2^, mean (SD)	108.5 (21.3)	108.5 (20.7)	108.5 (23.0)	0.998
LSM (kPa), median (IQR)	8.8 (7.3)	7.8 (3.8)	20.1 (10.4)	< 0.001
APRI score, median (IQR)	0.41 (0.52)	0.34 (0.33)	0.97 (1.25)	< 0.001
FIB-4 score, median (IQR)	0.88 (0.73)	0.81 (0.45)	1.69 (1.76)	< 0.001

SD: standard deviation; BMI: body mass index; IVDU: intravenous drug user; NRTI: nucleoside reverse transcriptase inhibitor; EFV: efavirenz; NVP: nevirapine; LPV/r: lopinavir/ritonavir; HCV-RNA: Hepatitis C virus-ribonucleic acid; ALT: alanine aminotransferase; AST: aspartate aminotransferase; eGFR: estimated glomerular filtration rate; CKD-EPI: chronic kidney disease-epidemiology collaboration; LSM: liver stiffness measurement; APRI: *AST* to *platelet ratio index;* FIB-4: the fibrosis-4 index; IQR: interquartile range; SD: standard deviation; kPa: kilo pascal

### Hepatitis C and liver fibrosis evaluation

In this study, mean HCV RNA viral load was 6.12 (SD 0.81) log_10_ IU/mL, as seen in Table 1. Median LSM for all the patients was 8.8 (IQR 7.3) kPa. Cirrhotic patients (28.7% of total participants) had median LSM 20.1 (IQR 10.4) kPa, while in non-cirrhotic patients the median LSM was 7.8 (IQR 3.8) kPa. Patients with cirrhosis had lower platelet count, higher ALT and AST level compared to non-cirrhotic patients. There was no difference between cirrhotic and non-cirrhotic patients in term of their HCV RNA viral load, hemoglobin, and kidney function (eGFR). The median values of fibrosis indices were: 0.41 (IQR 0.52) for APRI and 0.88 (IQR 0.73) for FIB-4. Higher values of APRI and FIB-4 were observed in patients with LSM ≥ 12.5 KPa compared to patients with LSM < 12.5 KPa: APRI 0.97 (IQR 1.25) vs 0.34 (IQR 0.33), *p* < 0.001 and FIB-4 1.69 (IQR 1.76) vs 0.81 (0.45), *p* < 0.001.

### Performance of APRI scores for the diagnosis of cirrhosis

**Fig. 1:**
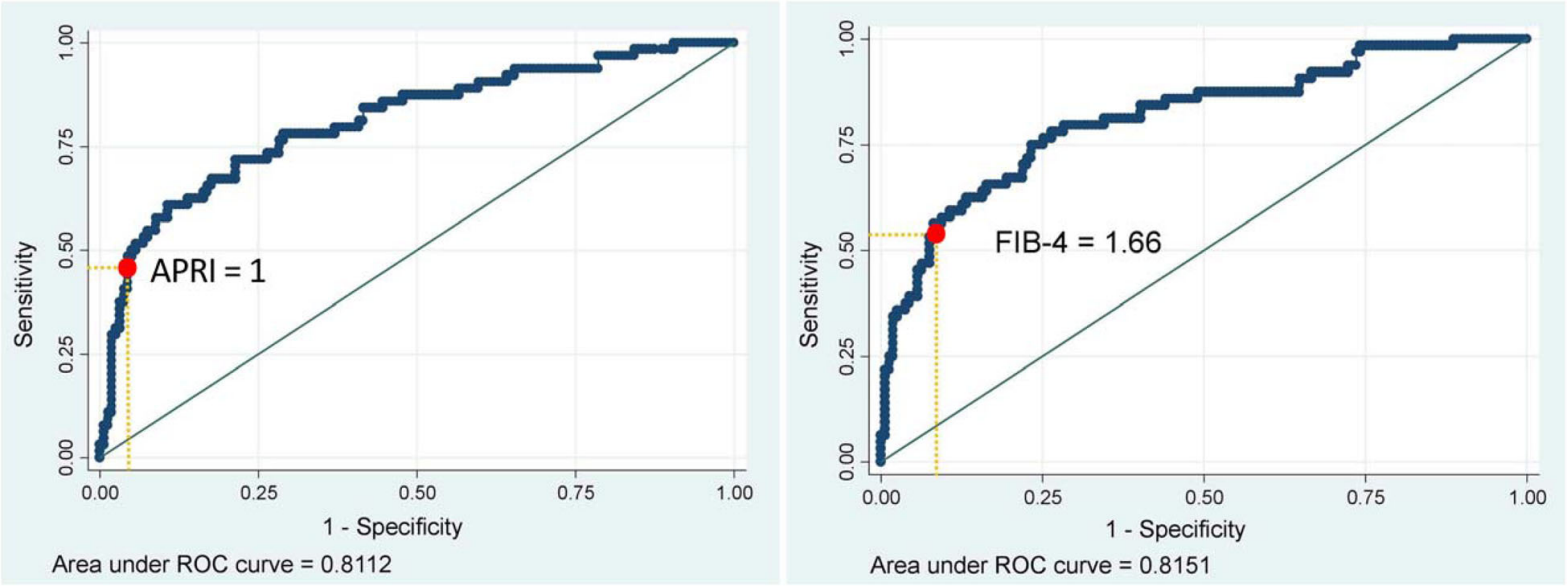
ROCs of transient elastography - APRI (left) and transient elastography - FIB-4 (right) of cirrhosis. ROC: receiving operating characteristic curve

We evaluated two different APRI threshold as recommended by WHO. In our population, an APRI value of 2 (high threshold) shown sensitivity 18.7%, specificity 98.1%, positive predictive value 80%, negative predictive value 75%, positive likelihood ratio 9.9 and negative likelihood ratio 0.8, with very poor performance [AUC 0.58 (95% CI 0.50–0.67)] and 75.3% were correctly classified. APRI value of 1 (low threshold) correctly classified 81.6% of patients and had sensitivity 48.4%, specificity 95%, positive predictive value 79.5%, negative predictive value 82.1%, positive likelihood ratio 9.6 and negative likelihood ratio 0.5 (see Table 2). This value had moderate performance with an AUC at 0.72 (95% CI 0.63–0.80) and revealed to be the optimal cut-off in defining cirrhosis in our patients. The respective ROC curves of APRI with the AUROC value is shown in Fig. 1.

**Table 2:** Performances of APRI and FIB-4 in predicting cirrhosis using transient elastography as a gold standard

	APRI 1	FIB-4 1.66
Se (95% CI)	48.4% (35.7–61.2)	53.1% (40.2–65.7)
Sp (95% CI)	95.0% (90.3–97.8)	92.5% (87.1–96.0)
PPV (95% CI)	79.5% (65.3–88.8)	73.9% (61.0–83.6)
NPV (95% CI)	82.1% (78.3–85.3)	83.1% (79.0–86.4)
LR+ (95% CI)	9.6 (4.7–19.8)	7.0 (3.9–12.7)
LR- (95% CI)	0.5 (0.4–0.7)	0.5 (0.3–0.6)
Ac (95% CI)	81.6% (75.9–86.5)	81.1% (75.4–86.0)
AUC (95% CI)	0.72 (0.63–0.80)	0.73 (0.65–0.81)

Se: sensitivity; Sp: specificity; PPV: positive predictive value; NPV: negative predictive value; LR-: negative likelihood ratio; LR+: positive likelihood ratio; Ac: accuracy; K: kappa; AUROC: area under the receiving operator curve

### Performance of FIB-4 scores for the diagnosis of cirrhosis

Using optimal cut-off of FIB-4 in previous studies, a value of 3.25 (high threshold) correctly classified 77.1% of patients with sensitivity 23.4%, specificity 98.7%, positive predictive value 88.2%, negative predictive value 76.2%, positive likelihood ratio 18.6 and negative likelihood ratio 0.8 in diagnosing liver cirrhosis. The performance was poor with an AUC at 0.61 (95% CI 0.52–0.70). Lower FIB-4 cut-off 1.94 correctly classified 79.3% of patients with sensitivity 37.5%, specificity 96.2%, positive predictive value 80%, negative predictive value 79.2%, positive likelihood ratio 9.9, and negative likelihood ratio 0.6. The performance of FIB-4 cut-off 1.94 was also poor with an AUC at 0.67 (95% CI 0.56–0.76). The optimal cut-off FIB-4 score for diagnosing cirrhosis in our patients was 1.66. As summarized in Table 2, at this cut-off, FIB-4 has sensitivity 53.1%, specificity 92.5%, positive predictive value 73.9%, negative predictive value 83.1%, positive likelihood ratio 7.0, and negative likelihood ratio 0.5. This FIB-4 new cut-off had better performance with AUC at 0.73 (95% CI 0.65–0.81) and 81.1% of patients were correctly classified. The ROC curves of FIB-4 with the AUROC value is shown in Fig. 1.

## DISSCUSSION

In our study, low threshold of APRI (1) demonstrate moderate performance [AUC 0.72 (95% CI 0.63–0.80)] in diagnosing cirrhosis. This cut-off was lower than cut-off use in Mazzola, et al. study (cut-off 2) with moderate performance (AUC 0,77) but almost similar with Merli, et al. study (cut off 0.97) with better performance [AUC 0.84 (95% 0.81–0.88)]. One third of population in the Merli, et al. study had cirrhosis as in our study, while prevalence of cirrhosis in Mazzola, et al. study was 49.6% [13, 14]. Low APRI threshold appeared to be better in diagnosis rather than detecting cirrhosis since the positive predictive value was good (79.5%), specificity was excellent (95%), and positive likelihood ratio was good (9.6). Using this threshold, 81.6% of the patients were correctly classified.

The optimal cut-off of FIB-4 in our population 1.66 was much lower than other studies. Merli, et al. used FIB-4 cut-off 2.02 and Mazzola, et al. used 5.88 for confirming cirrhosis [13, 14]. Our new cut-off shown a moderate performance with AUC 0.73 (95% CI 0.65–0.81). This new FIB-4 -cut-off was also better in diagnosis cirrhosis with the positive predictive value, specificity and positive likelihood ratio were good (73.9, 92.5%, 7.0, respectively). Using this new cut-off, we accurately classified cirrhosis and non-cirrhosis 81.1% of our population.

To achieve elimination goal, there is an urgent need to limit the cost for diagnostic procedure before starting HCV therapy, especially in resource-limited setting. In Indonesia only few TE machines are available, mainly located in big cities, and the cost is still high. Based on previous report from Indonesia, an average cost before starting HCV therapy was USD 191, including USD 74 for TE examination [2]. This high cost could delay treatment initiation for many patients though government provide the drugs. As the majority of HCV infection in Indonesia were driven by unsafe opiate injection around year 2000, we could predict that many untreated HCV-HIV co-infected patients have already in their cirrhosis stage [15, 16]. HIV co-infection could accelerate the developing of serious liver disease in one third of persons 20 years or less after infection [17]. About 15% of HCV advanced liver disease patients who did not initiate DAA treatment in the Chronic Hepatitis Cohort Study (CHeCS) had died [18].

Simple and accurate liver fibrosis assessment other than TE examination that can be done in remote area is essential [19, 20]. Since the Indonesian government planned to expand free DAA program for HCV, the result our study could be implemented in many places, as WHO also recommends to use APRI and FIB-4 in resource-constraint countries [2, 14].

This study has several limitations. First, the number of end-stage liver diseases were small. This may be caused by the recruited patients came from outpatient clinic where we rarely found end-stage liver disease stage. Quang Li, et al. demonstrated similar limitation among chronic infection of hepatitis B virus (HBV), the sample proportion were inappropriate, merely 7.2% cirrhotic-patient were cirrhosis [20]. Second, we did not use liver biopsy as gold standard in defining liver cirrhosis. However, our study actually shown a real-world data of TE comparison with these indirect biochemical markers, especially in Asia. Although TE is good validated in HCV infected patients, TE result could be interfered by confounding factors such as hepatic inflammation, hepatic congestion, extrahepatic cholestasis, and liver steatosis. Moreover, we did not investigate performance of biochemical markers against liver biopsy [19]. Third, we need to carefully interpret this result in female patients, since men represent majority of the study population (91.5%), as observed in other studies [14].

Fourth, the use of cut off of APRI and FIB-4 from this study could only be applied among compensated cirrhosis patients. Perez-Latorre, et al. mentioned decompensated cirrhosis patients have several activation of neurohormonal mechanism, leading to organ inflammatory damage, and decrease accuracy of TE [21]. Therefore, using TE in liver decompensated must be done carefully.

Fifth, we assessed the diagnostic performances of biochemical markers of fibrosis using TE as a gold standard although we could not validate its performances against liver biopsy in our study population. We recommend further cohort study and larger sample to get new optimal and valid cut-off of APRI and FIB-4.

## Conclusion

APRI and FIB-4 have moderate performance with high specificity in diagnosing cirrhosis. These biochemical markers could be used as diagnosis while TE is not available. In situation where TE is not available, our data support the use of these low-cost biochemical markers as diagnostic tools before DAA treatment.

## Data Availability

The data could be obtained by request to author’s email (evy.yunihastuti@gmail.com).

## Abbreviations

Ac: Accuracy
APRI: Aspartate aminotransferase to platelet ratio index
ART: Antiretroviral therapy
AUC: Area under curve
DAA: Direct acting antiviral
FIB-4: Fibrosis-4
GUCI: Göteborg university cirrhosis index
HBV: Hepatitis B virus
HCV: Hepatitis C virus
HIV: Human immunodeficiency virus
IVDU: Intravenous drug user
LR-: Negative likelihood ratio
LR +: Positive likelihood ratio.
LSM: Liver stiffness measurement
NPV: Negative predictive value
PPV: Positive predictive value
Se: Sensitivity
Sp: Specificity
SVR: Sustained virological response
TE: Transient elastography
WHO: World health organization
